# Natural convection heat transfer in an oscillating vertical cylinder

**DOI:** 10.1371/journal.pone.0188656

**Published:** 2018-01-05

**Authors:** Ilyas Khan, Nehad Ali Shah, Asifa Tassaddiq, Norzieha Mustapha, Seripah Awang Kechil

**Affiliations:** 1 Basic Engineering Sciences Department, College of Engineering Majmaah University, Majmaah, Saudi Arabia; 2 Abdus Salam School of Mathematical Sciences, GC University, Lahore, Pakistan; 3 College of Computer and Information Sciences, Majmaah University, Majmaah, Saudi Arabia; 4 Faculty of Computer and Mathematical Sciences, Universiti Teknologi MARA Kelantan, Machang, Kelantan, Malaysia; 5 Faculty of Computer and Mathematical Sciences, Universiti Teknologi MARA, UiTM Shah Alam, Malaysia; Vrije Universiteit Amsterdam, NETHERLANDS

## Abstract

This paper studies the heat transfer analysis caused due to free convection in a vertically oscillating cylinder. Exact solutions are determined by applying the Laplace and finite Hankel transforms. Expressions for temperature distribution and velocity field corresponding to cosine and sine oscillations are obtained. The solutions that have been obtained for velocity are presented in the forms of transient and post-transient solutions. Moreover, these solutions satisfy both the governing differential equation and all imposed initial and boundary conditions. Numerical computations and graphical illustrations are used in order to study the effects of Prandtl and Grashof numbers on velocity and temperature for various times. The transient solutions for both cosine and sine oscillations are also computed in tables. It is found that, the transient solutions are of considerable interest up to the times t = 15 for cosine oscillations and t = 1.75 for sine oscillations. After these moments, the transient solutions can be neglected and, the fluid moves according with the post-transient solutions.

## Introduction

Energy transfer due to convection is of great importance and arises in many physical situations [1]. Amongst the three different types of convections (free, forced, mixed), mixed convection is less investigated as compare to the other two types. When forced and free convections occur together, mixed convection induces. This phenomenon is usually seen in the channel flow due to heating or cooling of the channel walls. Energy transfer due to mixed convection is studied under different physical situations with various boundary constraints. For example, Fan, et al. [2] analyzed energy transfer because of mixed convection in a horizontal channel filled with nanofluids. Aaiza et al. [3, 4] examined energy transfer due to mixed convection in channel flow for ferrofluid and nanofluid respectively. Aaiza et al. [4], further pointed out that in mixed convection energy transfer, the buoyancy force is responsible for free convection and at least one of the two, non-homogeneous boundary conditions on velocity or external pressure gradient results forced convection. Amongst the important studies on mixed convection energy transfer, we include here the attempts those made by Kumari et al. [5], Tiwari and Das [6], Chamkha et al. [7], Sheikhzadeh et al. [8], Prasad et al. [9], Hasnain et al. [10] and Ganapathirao et al. [11]. However, most of these studies on energy transfer were focused in simple geometrical configurations.

In contrast, the energy transfer due to convection flow in stationary or moving cylinder has numerous applications in engineering and geophysics, such as nuclear reactor cooling system and underground energy transport and hence attracted the attention of many researchers. However, this area of research is not as much studied as flow over a flat plate, channel flow, flow over sheets etc. Most probably, it is due to complex nature of these problems. Most of these studies were investigated in the absence of heat or heat and mass transfer, see for example the work of Fetecau et al. [12–14], Jamil and Fetecau [15], Rubab et al. [16] and Abdulhameed et al. [17]. Such problems have also applications in biomagnetic fluid dynamics, see for example Sharma et al. [18], and Nehad et al. [19], where they used cylindrical coordinates and investigated the blood flow in cylindrical shaped arteries. Khan et al. [20–22], used cylindrical coordinates and investigated heat or heat and mass transfer in converging and diverging channels.

Free convection in cylindrical shape geometry is investigated in several earlier studies such as Goldstein and Briggs [23], in 1964 studied transient free convection over vertical plates and circular cylinders. Bottemanne [24] provided experimental results for pure and simultaneous heat and mass transfer by free convection over a vertical cylinder. Chen and Yuh [25] studied combined heat and mass transfer in free convection flow along a vertical cylinder. Some other related studies on free convection flow in a cylinder are given in [26–30]. In recent investigations, Deka et al. [31] analyzed transient free convection flow past an accelerated vertical cylinder in a rotating fluid whereas Deka and Paul [32] investigated unsteady one-dimensional free convection flow over an infinite moving vertical cylinder in the presence of thermal stratification. They used Laplace transform technique and obtained the exact solutions, expressed them in the forms of complicated integrals. Other interesting problems are studied in references [33–39].

The aim of this paper is to study the energy transfer in a vertically oscillating cylinder due to natural convection. Exact solutions are obtained by means of Laplace and Hankel transforms for velocity and temperature. The transient solutions for both cosine and sine oscillations of the cylinder are computed in tabular forms. Results of Prandtl and Grashof numbers for different times are shown in graphs and discussed.

## Mathematical formulation and solution of the problem

**Fig 1:**
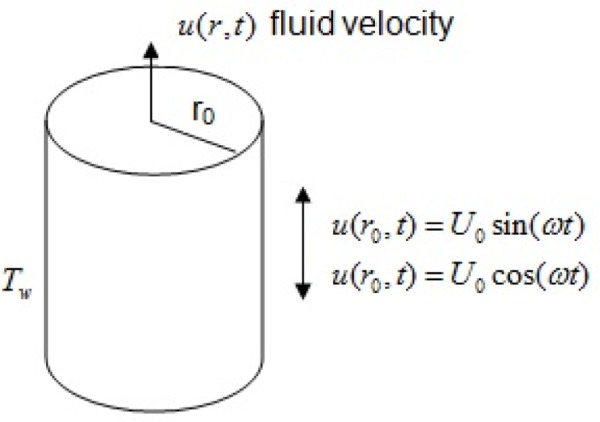
Fluid flow geometry.

Let us consider transient free convection flow of an incompressible viscous fluid in an infinite vertical cylinder of radius *r*_0_. The z-axis is considered along the axis of cylinder in vertical upward direction and the radial coordinate *r* is taken normal to it. Initially at time *t* ≤ 0, it is assumed that the cylinder is at rest and the cylinder and fluid are at the same temperature *T*_∞_. After time *t* = 0, the cylinder begins to oscillate along its axis and induces the motion in the fluid with velocity *U*_0_*H*(*t*)exp(*iωt*), where *U*_0_ is the characteristic velocity, *H*(*t*) is the unit step function and *ω* is the frequency of oscillation. At the same time, the cylinder temperature raised to *T*_*w*_ which is thereafter maintained constant (Fig 1). We assume that the velocity and temperature are the function of *r* and *t* only. For such a flow, the constraint of incompressibility is identically satisfied. It is also assumed that all the fluid properties are constant except for the density in the buoyancy term, which is given by the usual Boussinesq’s approximation. In this paper, we have proposed to obtain analytical solutions for the temperature and velocity fields, in the negligible dissipation hypothesis. Under these assumptions, a well-defined problem is modeled in terms of the following partial differential equations:

$$\frac{\partial^{2}u\left(r,t\right)}{\partial r^{2}}+\frac{1}{r}\frac{\partial u\left(r,t\right)}{\partial r}-\frac{1}{\nu }\frac{\partial u\left(r,t\right)}{\partial t}+\frac{g\beta_{T}}{\nu }(T\left(r,t\right)-T_{\infty })=0\;;\;r\in (0,r_{0}),t>0,\;$$ (1)

$$\frac{\partial^{2}T\left(r,t\right)}{\partial r^{2}}+\frac{1}{r}\frac{\partial T\left(r,t\right)}{\partial r}-\frac{1}{\alpha }\;\frac{\partial T\left(r,t\right)}{\partial t}=0\;;\;r\in (0,\;r_{0}),t>0\;,$$ (2)

with appropriate initial and boundary conditions:

$$u\left(r,0\right)=0,\;\;T(r,0)=T_{\infty }\;;\;r\in [0,\;r_{0}],\;$$ (3)

$$u\left(r_{0},t\right)=U_{0}H(t)\exp(i\omega t);\;T(r_{0},t)=T_{w},\;t>0\;.$$ (4)

Introducing the following dimensionless variables:

$$t^{*}=\frac{t\nu }{r_{0}^{2}},\;r^{*}=\frac{r}{r_{0}},\;u^{*}=\frac{u}{U_{0}},\;\theta =\frac{T-T_{\infty }}{T_{w}-T_{\infty }},\;\omega^{*}=\frac{\omega r_{0}^{2}\;}{\nu },$$ (5)

the governing Eqs (1)–(4) reduce to (dropping out the star notation):

$$\frac{\partial u\left(r,t\right)}{\partial t}=\frac{\partial^{2}u\left(r,t\right)}{\partial r^{2}}+\frac{1}{r}\frac{\partial u\left(r,t\right)}{\partial r}+Gr\theta \;;\;r\in (0,1),\;t>0,\;$$ (6)

$$\frac{\partial \theta \left(r,t\right)}{\partial t}=\frac{1}{\mathrm{Pr}}\left(\frac{\partial^{2}\theta \left(r,t\right)}{\partial r^{2}}+\frac{1}{r}\frac{\partial \theta \left(r,t\right)}{\partial r}\right)\;;\;r\in (0,1),t>0\;,\;$$ (7)

u(r,0)=0,θ(r,0)=0;r∈[0,1], (8)

u(1,t)=H(t)exp(iωt);θ(1,t)=1,t>0, (9)

where $\;Gr=\frac{g\beta_{T}r_{0}^{2}(T_{w}-T_{\infty })}{U_{0}\nu }$, $\mathrm{Pr}=\frac{\nu }{\alpha }$.

### Calculation for temperature

Applying the Laplace transform to Eqs (7), (9)_2_ and using the initial condition (8)_2_, we obtain the following transformed problem:

$$q\bar{\theta }(r,\;q)=\frac{1}{\mathrm{Pr}}\left(\frac{\partial^{2}}{\partial r^{2}}+\frac{1}{r}\frac{\partial }{\partial r}\right)\bar{\theta }(r,\;q),$$ (10)

$$\bar{\theta }(1,q)=\frac{1}{q},$$ (11)

where $\bar{\theta }(r,q)$ is the Laplace transform of the function *θ*(*r*,*t*) and *q* is the transform variable.

Applying the finite Hankel transform of order zero, to Eq (10), and using condition (11), we obtain:

$${\bar{\theta }}_{H}(r_{n},\;q)=\frac{J_{1}(r_{n})}{r_{n}}\left(\frac{1}{q}-\frac{1}{q+\frac{r_{n}^{2}}{\mathrm{Pr}}}\right),$$ (12)

where ${\bar{\theta }}_{H}(r_{n},\;q)=\int_{0}^{1}r\bar{\theta }(r,\;q)J_{0}(rr_{n})dr$ is the finite Hankel transform of the function $\bar{\theta }(r,q)$ *r*_*n*_, *n* = 0,1,… are the positive roots of the equation *J*_0_(*x*) = 0, *J*_0_ being the Bessel function of first kind and zero order.

Taking inverse Laplace transform of Eq (12), we obtain:

$$\theta_{H}(r_{n},t)=\frac{J_{1}(r_{n})}{r_{n}}-\frac{J_{1}(r_{n})}{r_{n}}\exp\left(-\frac{r_{n}^{2}}{\mathrm{Pr}}t\right).$$ (13)

Taking inverse Hankel transform, we obtain

$$\theta (r,t)=1-2\sum_{n=1}^{\infty }\frac{J_{0}(rr_{n})}{r_{n}J_{1}(r_{n})}\exp\left(-\frac{r_{n}^{2}}{\mathrm{Pr}}t\right).$$ (14)

In order to study the heat transfer from the cylinder surface to the fluid, we determine the Nusselt number. This dimensionless number is defined as ratio of the convective heat transfer to the conductive heat transfer and is given by

$$Nu=-{\left(\frac{\partial \theta (r,t)}{\partial r}\right)}_{r=1}=2\sum_{n=1}^{\infty }\exp\left(-\frac{r_{n}^{2}}{\mathrm{Pr}}t\right).$$ (15)

### Calculation for velocity

Applying the Laplace transform to Eqs (6), (9)_1_, and using the initial condition (8)_1_, we obtain

$$q\bar{u}(r,q)=\frac{\partial^{2}\bar{u}(r,q)}{\partial r^{2}}+\frac{1}{r}\frac{\partial \bar{u}(r,q)}{\partial r}+Gr\bar{\theta }(r,q),$$ (16)

$$\bar{u}(1,q)=\frac{1}{q-i\omega }.$$ (17)

Applying finite Hankel transform to Eq (16) and using Eqs (12), (17), we have

$${\bar{u}}_{H}(r_{n},q)=\frac{1}{\left(q-i\omega )(q+r_{n}^{2}\right)}r_{n}J_{1}(r_{n})+Gr\frac{J_{1}(r_{n})}{r_{n}}\left[\frac{1}{q(q+r_{n}^{2})}-\frac{1}{(q+r_{n}^{2})\left(q+\frac{r_{n}^{2}}{\mathrm{Pr}}\right)}\right],$$ (18)

where ${\bar{u}}_{H}(r_{n},\;q)=\int_{0}^{1}r{\bar{u}}_{H}(r,\;q)J_{0}(rr_{n})dr$ is the finite Hankel transform of the function $\bar{u}(r,q).$

We consider

$$F_{n}(q)=\frac{r_{n}J_{1}(r_{n})}{\left(q-i\omega )(q+r_{n}^{2}\right)}=\frac{r_{n}J_{1}(r_{n})}{r_{n}^{2}+i\omega }\frac{1}{q-i\omega }-\frac{r_{n}J_{1}(r_{n})}{r_{n}^{2}+i\omega }\frac{1}{q+r_{n}^{2}}=F_{1n}(q)+F_{2n}(q),$$ (19)

where

$$F_{1n}(q)=\frac{r_{n}J_{1}(r_{n})}{r_{n}^{2}+i\omega }\frac{1}{q-i\omega }=\frac{J_{1}(r_{n})}{r_{n}}\frac{1}{q-i\omega }-\frac{J_{1}(r_{n})}{r_{n}}\frac{\omega (\omega +ir_{n}^{2})}{r_{n}^{4}+\omega^{2}}\frac{1}{q-i\omega },$$ (20)

$$F_{2n}(q)=-\frac{r_{n}J_{1}(r_{n})}{r_{n}^{2}+i\omega }\frac{1}{q+r_{n}^{2}}=-\frac{r_{n}J_{1}(r_{n})(r_{n}^{2}-i\omega )}{r_{n}^{4}+\omega^{2}}\frac{1}{q+r_{n}^{2}},$$ (21)

$$F_{3n}(q)=\frac{GrJ_{1}(r_{n})}{r_{n}}\left[\frac{1}{r_{n}^{2}}\left(\frac{1}{q}-\frac{1}{q+r_{n}^{2}}\right)-\frac{\mathrm{Pr}}{r_{n}^{2}(1-\mathrm{Pr})}\left(\frac{1}{q+r_{n}^{2}}-\frac{1}{q+\frac{r_{n}^{2}}{\mathrm{Pr}}}\right)\right].$$ (22)

Applying the inverse Laplace transform to Eqs (19), (20), (21) and (22), we obtain

$$f_{n}(t)=f_{1n}(t)+f_{2n}(t),$$ (23)

with

$$\begin{array}{c} f_{1n}(t)=\frac{J_{1}(r_{n})}{r_{n}}\exp(i\omega t)-\omega^{2}\cos(\omega t)\frac{J_{1}(r_{n})}{r_{n}(r_{n}^{4}+\omega^{2})}+\omega \sin(\omega t)\frac{r_{n}J_{1}(r_{n})}{\left(r_{n}^{4}+\omega^{2}\right)}-\\ \;-i\left[\omega \cos(\omega t)\frac{r_{n}J_{1}(r_{n})}{\left(r_{n}^{4}+\omega^{2}\right)}+\omega^{2}\sin(\omega t)\frac{J_{1}(r_{n})}{r_{n}(r_{n}^{4}+\omega^{2})}\right], \end{array}$$ (24)

$$f_{2n}(t)=-\frac{r_{n}^{3}J_{1}(r_{n})}{r_{n}^{4}+\omega^{2}}\exp(-r_{n}^{2}t)+i\frac{\omega r_{n}J_{1}(r_{n})}{r_{n}^{4}+\omega^{2}}\exp(-r_{n}^{2}t)$$ (25)

$$f_{3n}(t)=\frac{GrJ_{1}(r_{n})}{r_{n}^{3}}+\frac{GrJ_{1}(r_{n})}{r_{n}^{3}(\mathrm{Pr}-1)}\exp(-r_{n}^{2}t)-\frac{Gr\mathrm{Pr}J_{1}(r_{n})}{r_{n}^{3}(\mathrm{Pr}-1)}\exp\left(-\frac{r_{n}^{2}t}{\mathrm{Pr}}\right),\;\mathrm{Pr}\neq 1.$$ (26)

Applying the Laplace transform to Eq (18) and using Eqs (23)–(26), we obtain

$$\begin{array}{l} u_{H}(r_{n},\;t)=\frac{J_{1}(r_{n})}{r_{n}}\exp(i\omega t)-\omega^{2}\cos(\omega t)\frac{J_{1}(r_{n})}{r_{n}(r_{n}^{4}+\omega^{2})}+\omega \sin(\omega t)\frac{r_{n}J_{1}(r_{n})}{\left(r_{n}^{4}+\omega^{2}\right)}-\\ \;-i\left[\omega \cos(\omega t)\frac{r_{n}J_{1}(r_{n})}{\left(r_{n}^{4}+\omega^{2}\right)}+\omega^{2}\sin(\omega t)\frac{J_{1}(r_{n})}{r_{n}(r_{n}^{4}+\omega^{2})}\right]-\frac{r_{n}^{3}J_{1}(r_{n})}{r_{n}^{4}+\omega^{2}}\exp(-r_{n}^{2}t)+i\frac{\omega r_{n}J_{1}(r_{n})}{r_{n}^{4}+\omega^{2}}\exp(-r_{n}^{2}t)+\\ \;+\frac{GrJ_{1}(r_{n})}{r_{n}^{3}}+\frac{GrJ_{1}(r_{n})}{r_{n}^{3}(\mathrm{Pr}-1)}\exp(-r_{n}^{2}t)-\frac{Gr\mathrm{Pr}J_{1}(r_{n})}{r_{n}^{3}(\mathrm{Pr}-1)}\exp\left(-\frac{r_{n}^{2}t}{\mathrm{Pr}}\right),\;\mathrm{Pr}\neq 1.\; \end{array}$$ (27)

Applying the inverse Hankel transform to Eq (27), we obtain:

$$\begin{array}{c} u(r,\;t)=\exp(i\omega t)-2\omega^{2}\cos(\omega t)a_{1}(r)+2\omega \sin(\omega t)b_{1}(r)-2\sum_{n=1}^{\infty }\frac{r_{n}^{3}J_{0}(rr_{n})}{\left(r_{n}^{4}+\omega^{2}\right)J_{1}(r_{n})}\exp(-r_{n}^{2}t)+\\ \;+\frac{2Gr}{\mathrm{Pr}-1}\sum_{n=1}^{\infty }\left[\left((\mathrm{Pr}-1)+\exp(-r_{n}^{2}t)-\mathrm{Pr}\;\exp\left(-\frac{r_{n}^{2}t}{\mathrm{Pr}}\right)\right)\frac{J_{0}(rr_{n})}{r_{n}^{3}J_{1}(r_{n})}\right]-\\ \;-i\left[2\omega \cos(\omega t)b_{1}(r)+2\omega^{2}\sin(\omega t)a_{1}(r)-2\omega \sum_{n=1}^{\infty }\frac{r_{n}J_{0}(rr_{n})\exp(-r_{n}^{2}t)}{\left(r_{n}^{4}+\omega^{2})J_{1}(r_{n}\right)}\right],\;\mathrm{Pr}\neq 1,\; \end{array}$$ (28)

where $a_{1}(r)=\sum_{n=1}^{\infty }\frac{J_{0}(rr_{n})}{r_{n}(r_{n}^{4}+\omega^{2})J_{1}(r_{n})}$ and $b_{1}(r)=\sum_{n=1}^{\infty }\frac{r_{n}J_{0}(rr_{n})}{\left(r_{n}^{4}+\omega^{2})J_{1}(r_{n}\right)}$.

### Cosine oscillation

For cosine oscillations of cylinder, the velocity field is given as:

$$u_{c}(r,\;t)=u_{cp}(r,\;t)+u_{ct}(r,\;t),$$ (29)

with

$$u_{cp}(r,\;t)=[1-2\omega^{2}a_{1}(r)]\cos(\omega t)+2\omega b_{1}(r)\sin(\omega t)+2Gr\sum_{n=1}^{\infty }\frac{J_{0}(rr_{n})}{r_{n}^{3}J_{1}(r_{n})},$$ (30)

$$u_{ct}(r,\;t)=\frac{2Gr}{\mathrm{Pr}-1}\sum_{n=1}^{\infty }\left[\frac{J_{0}(rr_{n})}{r_{n}^{3}J_{1}(r_{n})}\left(\exp(-r_{n}^{2}t)-\mathrm{Pr}\;\exp\left(-\frac{r_{n}^{2}t}{\mathrm{Pr}}\right)\right)\right]-2\sum_{n=1}^{\infty }\frac{r_{n}^{3}J_{0}(rr_{n})}{\left(r_{n}^{4}+\omega^{2}\right)J_{1}(r_{n})}\exp(-r_{n}^{2}t),$$ (31)

are the permanent solution, respectively, the transient solution of cosine oscillation.

### Sine oscillation

For sine oscillations of cylinder, the velocity field is given as:

$$u_{s}(r,\;t)=u_{sp}(r,\;t)+u_{st}(r,\;t),$$ (32)

with

$$u_{sp}(r,\;t)=[1-2\omega^{2}a_{1}(r)]\sin(\omega t)+2\omega b_{1}(r)\cos(\omega t),$$ (33)

$$u_{st}(r,\;t)=2\omega \sum_{n=1}^{\infty }\left[\frac{r_{n}J_{0}(rr_{n})}{\left(r_{n}^{4}+\omega^{2})J_{1}(r_{n}\right)}\exp(-r_{n}^{2}t)\right],$$ (34)

are the permanent solution, respectively, the transient solution of sine oscillation.

## Numerical results and discussions

**Fig 2:**
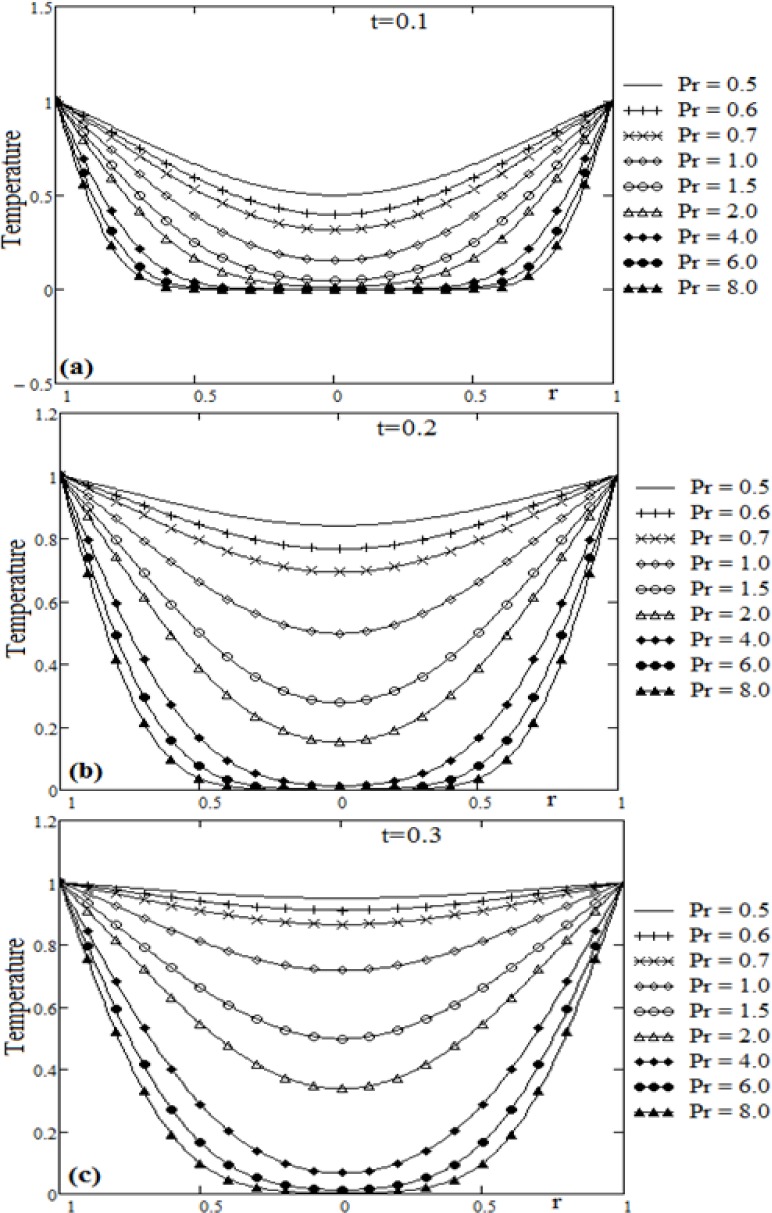
Profiles of temperature for Prandtl number Pr variation and different values of time t.

**Fig 3:**
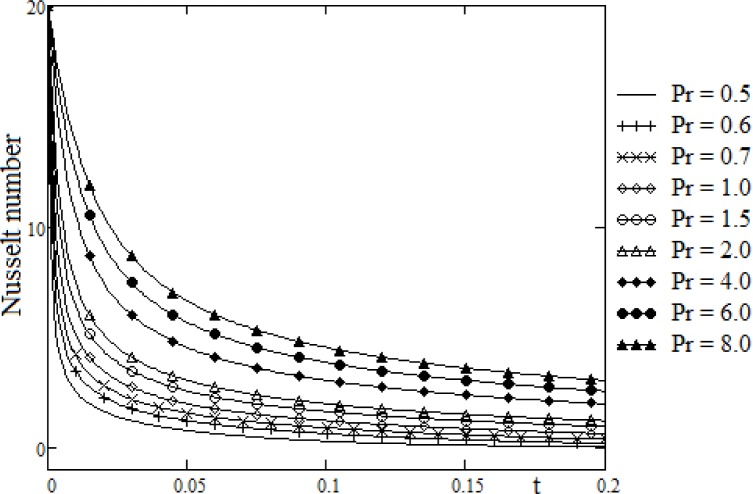
Variation of Nusselt number for different values of Pr.

**Fig 4:**
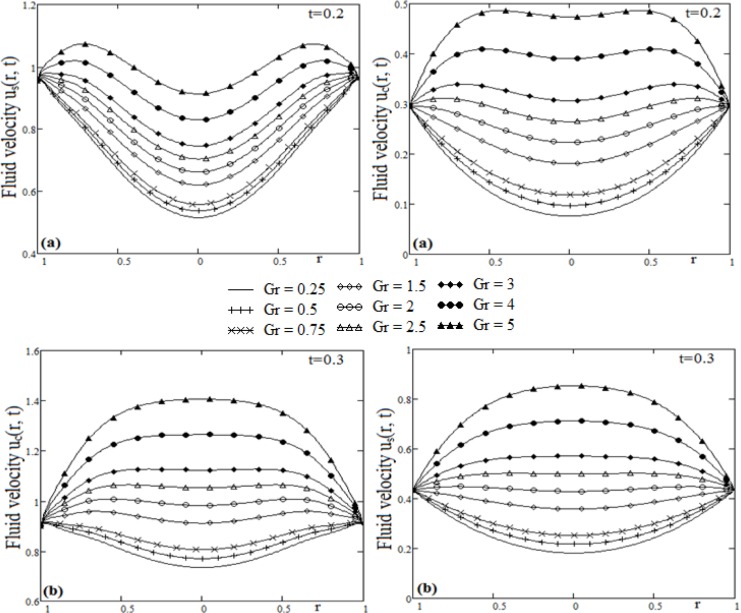
Profiles of velocity for cosine and since oscillations for Grashof number Gr variation and different time t.

**Fig 5:**
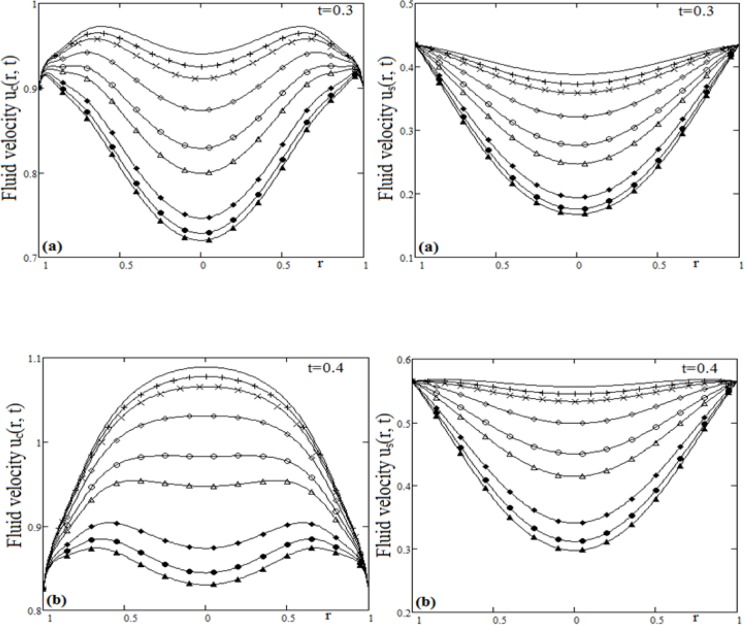
Profiles of velocity for cosine and since oscillations for Prandtl number Pr variation and different time t.

In order to obtain some information on the fluid flow parameters and heat transfer, we have made numerical simulations using Mathcad software. The obtained results are presented in the graphs from Figs 2–5. Geometry of the problem is given in Fig 1.

We were interested, to analyze the influence of the Prandtl number on the temperature, Nusselt number and on fluid velocity. Also, the influence of the Grashof number on the fluid velocity was studied.

To evaluate numerical values of the temperature, Nusselt number and of the fluid velocity, we need the positive roots of the Bessel function *J*_0_. These roots are generated by a numerical subroutine using Mathcad. All the parameters and profiles are dimensionless.

The diagrams of Fig 2 is plotted in order to discuss the influence of the Prandl number Pr, on the fluid temperature. The curves corresponding to the dimensionless temperature *θ*(*r*,*t*), are sketched versus the radial coordinate *r*, for different values of the time t and Prandtl number Pr. It is clear from the temperature expression (14) that, the exponential term tends fast to zero for large time or, for small values of the Prandtl number. This is due to the fast increasing values of the positive roots of the function J0(x). It is observed from Fig 2 that, for values of the Prandtl number greater than 2, the fluid situated in the central area of the cylinder is not heated for small values of the time t. For small values of the Prandtl number the heat transfer from the cylinder surface to fluid is significant. Decrease in Prandtl number implies thickening of thermal boundary layer, therefore, the temperature gradient decreases with Prandtl.

Fig 3 shows the diagrams of the local Nusselt number Nu for different values of the Prandtl number Pr. The results are depicted versus time variable t. The remarkable effect of the Prandtl number is clear. When the number Pr is increased, the Nusselt number is increasing. At small values of the time t, the Nusselt number has big values, which means that, for small values of time t, the convection is very efficient. For large values of the time t, the conduction is dominant and, the heat transfer is produced only by conduction for very large values of the time t (for *t* → ∞, the Nusselt number tends to zero).

The influence of Grashof number Gr, on the fluid velocity is shown in Fig 4. To draw the curves from Fig 4, we used values Pr = 0.7 and ω = 1.5. It must be emphasized that, for small values of Grashof number the fluid velocity has low values than in the case of large values of the Grashof number. This is due to the contribution of the temperature in the fluid velocity solution. Low values of the Grashof number lead to reduced contribution of the temperature in the fluid flow, therefore, the viscous forces increase and the velocity decreases.

Fig 5, was drawn in order to analyze the influence of the Prandtl number Pr, on the fluid velocity. Both cases of the plate oscillations were considered, namely cosine oscillations and sine oscillations. For Fig 5, we used values Gr = 1.5 and ω = 1.5. The buoyancy forces created by the density differences are high for the smaller values of the Prandtl number when the temperature is high. When the Prandtl number is large, the viscous damping action becomes bigger and fluid velocity decreases.

The decreasing of the transient solution *u*_*ct*_(*r*,*t*), given by Eq (31), is shown in the Table 1, for Gr = 5, Pr = 7 and *ω* = 0.449. It is observed from Table 1 that, for t = 15 the transient solution *u*_*ct*_(*r*,*t*), is of order 10^−6^, therefore, after this moment the transient solution can be neglected and, the fluid moves according with the post-transient solution.

**Table 1:** Degreasing of the transient solution *u*_*ct*_(*r*,*t*), with the time *t*, for *Gr* = 5, Pr = 7 and *ω* = 0.449.

*r*	*u*_*ct*_(*r*,0.5)	*u*_*ct*_(*r*,5)	*u*_*ct*_(*r*,15)
0	-0.60508	-0.01298	-3.35246×10^−6^
0.1	-0.59704	-0.0128	-3.30416×10^−6^
0.2	-0.57311	-0.01224	-3.16136×10^−6^
0.3	-0.53393	-0.01135	-2.93022×10^−6^
0.4	-0.48063	-0.01015	-2.62065×10^−6^
0.5	-0.41489	-8.69762×10^−3^	-2.24591×10^−6^
0.6	-0.33902	-7.05552×10^−3^	-1.82188×10^−6^
0.7	-0.25595	-5.29157×10^−3^	-1.36639×10^−6^
0.8	-0.16916	-3.47889×10^−3^	-8.98323×10^−6^
0.9	-0.08252	-1.69133×10^−3^	-4.36737×10^−6^
1	0	0	0

Similarly, in Table 2 is presented the decreasing with time t of the transient solution corresponding to the sine oscillations of the cylinder, given by Eq (34). Comparing with the cosine oscillations, it is seen that, the critical time at which the transient solution is of order 10^−6^ is lower for sine oscillations. For the same values of the system parameters, the transient solution for sine oscillations can be neglected after the value t = 1.75.

**Table 2:** Degreasing of the transient solution *u*_*st*_(*r*,*t*), with the time *t*, for *Gr* = 5, Pr = 7 and *ω* = 0.449.

*r*	*u*_*st*_(*r*,0.1)	*u*_*st*_(*r*,1)	*u*_*st*_(*r*,1.75)
0	0.06856	3.80476×10^−4^	4.97305×10^−6^
0.1	0.06762	3.74995×10^−4^	4.90141×10^−6^
0.2	0.06482	3.58788×10^−4^	4.68958×10^−6^
0.3	0.06026	3.32555×10^−4^	4.34669×10^−6^
0.4	0.0541	2.97422×10^−4^	3.88749×10^−6^
0.5	0.04655	2.54892×10^−4^	3.33159×10^−6^
0.6	0.03792	2.06769×10^−4^	2.70259×10^−6^
0.7	0.02855	1.55074×10^−4^	2.02692×10^−6^
0.8	0.01882	1.01952×10^−4^	1.33258×10^−6^
0.9	0.16941×10^−3^	4.9566×10^−5^	6.47858×10^−7^
1	0	0	0

## Conclusions

The problem of heat transfer due to free convection in an oscillating vertical cylinder is studied. Exact solutions for temperature and velocity are determined by applying the Laplace and finite Hankel transforms. The velocity solutions are arranged in transient and post-transient parts. Obtained analytical results were plotted and discussed. Transient solutions were computed in tables. The main points of this study are listed below:

- Increasing Prandtl number Pr, the temperature decreases. The Nusselt number increases if the Prandtl number increases.
- For small values of the time t, the convection is dominant, while, for large values of time t the heat transfer by conduction is dominant.
- Fluid velocity increasing with Grashof number but decreasing with Prandtl number.
- The transient solutions are significant up to the order of 10^−6^, and thereafter the fluid moves according with the post-transient solutions.
